# Linking Antibodies Against Apolipoprotein A-1 to Metabolic Dysfunction-Associated Steatohepatitis in Mice

**DOI:** 10.3390/ijms252211875

**Published:** 2024-11-05

**Authors:** Sabrina Pagano, Emmanuel Somm, Catherine Juillard, Nicolas Liaudet, Frédérique Ino, Johan Ferrari, Vincent Braunersreuther, François R. Jornayvaz, Nicolas Vuilleumier

**Affiliations:** 1 Division of Laboratory Medicine, Diagnostic Department, Geneva University Hospitals, 1211 Geneva, Switzerland; nicolas.vuilleumier@hug.ch; 2 Department of Medicine, Medical Faculty, Geneva University, 1211 Geneva, Switzerland; catherine.juillard@unige.ch; 3 Service of Endocrinology, Diabetes, Nutrition and Therapeutic Patient Education, Department of Internal Medicine, Geneva University Hospitals, 1211 Geneva, Switzerland; emmanuel.somm@unige.ch (E.S.); frederique.ino@unige.ch (F.I.); francois.jornayvaz@hug.ch (F.R.J.); 4 Department of Cell Physiology and Metabolism, University of Geneva, 1211 Geneva, Switzerland; 5 Diabetes Center, the Faculty of Medicine, University of Geneva, 1211 Geneva, Switzerland; 6 Bioimaging Core Facility, Medical Faculty, University of Geneva, 1211 Geneva, Switzerland; nicolas.liaudet@unige.ch; 7 Division of Clinical Pathology, Diagnostic Department, Geneva University Hospitals, 1211 Geneva, Switzerland; johan.ferrari@hug.ch (J.F.); vincent.braunersreuther@hug.ch (V.B.)

**Keywords:** anti-apolipoprotein A1 antibodies, MASLD, MASH, CDAHFD mouse, Cytokeratin 18, inflammation

## Abstract

Metabolic dysfunction-associated fatty liver disease (MASLD) is a common liver and health issue associated with heightened cardiovascular disease (CVD) risk, with Cytokeratin 18 (CK-18) as a marker of liver injury across the MASLD to cirrhosis spectrum. Autoantibodies against apolipoprotein A-1 (AAA-1s) predict increased CVD risk, promoting atherosclerosis and liver steatosis in apoE−/− mice, though their impact on liver inflammation and fibrosis remains unclear. This study examined AAA-1s’ impact on low-grade inflammation, liver steatosis, and fibrosis using a MASLD mouse model exposed to AAA-1s passive immunization (PI). Ten-week-old male C57BL/6J mice under a high-fat diet underwent PI with AAA-1s or control antibodies for ten days. Compared to controls, AAA-1-immunized mice showed higher plasma CK-18 (5.3 vs. 2.1 pg/mL, *p* = 0.031), IL-6 (13 vs. 6.9 pg/mL, *p* = 0.035), IL-10 (27.3 vs. 9.8 pg/mL, *p* = 0.007), TNF-α (32.1 vs. 24.2 pg/mL, *p* = 0.032), and liver steatosis (93.4% vs. 73.8%, *p* = 0.007). Transcriptomic analyses revealed hepatic upregulation of pro-fibrotic mRNAs in AAA-1-recipient mice, though histological changes were absent. In conclusion, short-term AAA-1 PI exacerbated liver steatosis, inflammation, and pro-fibrotic gene expression, suggesting that AAA-1s may play a role in MASLD progression. Further research with prolonged AAA-1 exposure is warranted to clarify their potential role in liver fibrosis and associated complications.

## 1. Introduction

Metabolic dysfunction-associated fatty liver disease (MASLD) and its advanced stage of metabolic dysfunction-associated steatohepatitis (MASH), previously known as NAFLD and NASH, respectively, are complex systemic metabolic disorders [1]. Initially considered as a liver-restricted pathology only, MASLD is nowadays viewed as a multi-systemic condition affecting numerous organs, such as adipose tissue, muscle, and intestine with important consequences on the renal and cardiovascular system [2,3]. The annual medical costs directly linked to MASLD are estimated to exceed EUR 35 billion in Europe and USD 100 billion in the United States [4].

One of the most widely investigated biomarkers for MASH diagnosis in patients with MASLD is circulating keratin 18 (CK-18) fragments. Their release in the circulation is believed to reflect cytoskeleton injury occurring upon hepatocellular ballooning, the hallmark of steatohepatitis [5,6,7]. Covering the whole disease spectrum from MASLD to cirrhosis [5,6,7], CK-18 levels of elevation are also observed in diseases with increased cardiovascular (CV) risk, such as chronic kidney disease [8], type 2 diabetes (T2D) [9], and other conditions related to endoplasmic reticulum and oxidative stress [10,11]. Several studies highlighted CK-18 as being positively associated with cardiometabolic disorders and CV risk [12,13,14] as well as with coronary artery disease severity and systolic dysfunction after acute myocardial infarction [15,16].

Autoantibodies against apolipoprotein A-1 (AAA-1s) are known to predict poor CV prognosis in general population individuals [17] and in high CV-risk patients [18,19,20,21,22]. In addition, these autoantibodies were found to be raised in obese subjects where they predict the presence of coronary calcification lesions [23]. In metabolic syndrome patients undergoing a Mediterranean diet, AAA-1s were associated with resistance to waist circumference reduction [24] and decreased excess body mass index loss after bariatric surgery [25].

Moreover, AAA-1s play a direct pathogenic role as pro-inflammatory and dyslipidemic mediators through multiple cellular pathways (increase in intracellular cholesterol synthesis and lipid uptake, decreased cellular membrane lipid passive diffusion) to culminate into macrophage foam cell formation [26]. Finally, AAA-1s were also shown to promote hepatic steatosis through triglyceride pathway disruption, a key step in hepatic steatosis and MAFLD [27]. With such biological properties, interactions between AAA-1s, MASLD, and CVD are expected, especially because the association between AAA-1s and increased 10-year CV risk (according to the Framingham risk score) in the general population was shown to be dependent on the fatty liver index [27].

Numerous mouse models of MASLD have been developed so far. While apoE−/− mice on a Western diet have proven to be a convenient model for advanced NASH/MASH [28,29], mice fed a choline-deficient, L-amino-acid-defined, high-fat diet (CDAHFD) are considered to be an earlier MASH model due to the absence of fibrosis within one week of the diet despite developing steatosis and steatohepatitis more rapidly and severely than conventional models [30].

Therefore, the aim of our study was to replicate the ability of AAA-1 passive immunization (PI) to enhance hepatic steatosis in CDAHFD mice and investigate its impact on steatohepatitis and early liver fibrosis.

## 2. Results

### 2.1. Treatment with AAA-1s Promote Steatohepatitis

**Figure 1:**
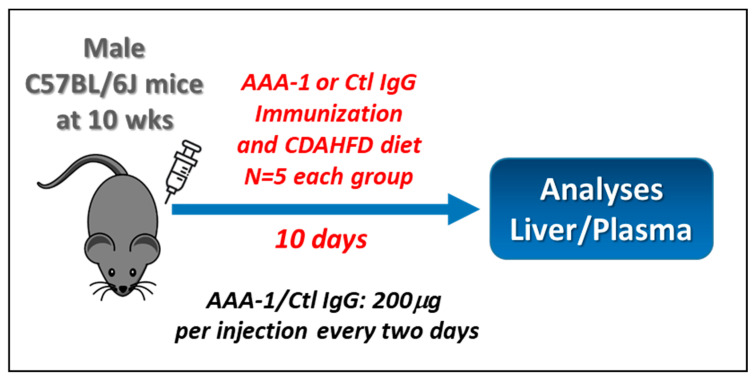
Overview of the experimental approach.

The overview of the animal experiment is shown in Figure 1. Ten-week-old C57BL/6J mice were fed a CDAHFD diet for ten days and were simultaneously divided into two groups to receive either AAA-1s or control IgG (Ctl IgG) antibodies for ten days to identify possible differences in AAA-1s ability to prime steatohepatitis.

**Figure 2:**
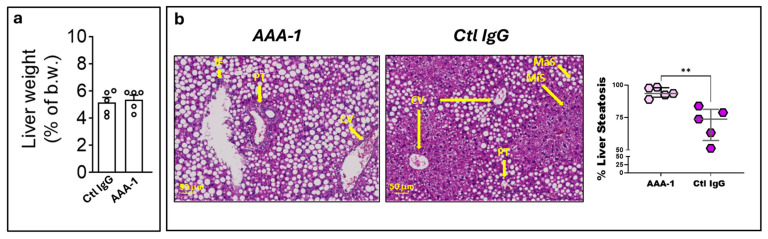
Aggravation of hepatic steatosis in CDAHFD mice by AAA-1 immunization. (**a**) No difference in liver weight was observed between the two groups after the PI protocol. Results are presented as individual values, with bars indicating the mean and standard deviation (**b**) The abnormal accumulation of fat in hepatic steatosis appears on H&E staining as clear vacuoles within hepatocytes, displacing the nucleus to the cell’s periphery. These vacuoles, formed by lipid droplets that do not stain with H&E, appear as clear spaces in the pink-stained cytoplasm. Representative H&E staining of liver sections from CDAHFD mice immunized with AAA-1s or Ctl IgG. ** *p*= 0.007. Mann–Whitney (M-W) test. The results are presented as median and interquartile range. Yellow arrows indicate liver structures. Abbreviations, IF: inflammatory foci; PT: portal triad; CV: centrilobular vein; MaS: macrosteatosis; MiS: microsteatosis.

Upon sacrifice, no differences in liver weight were observed between the two groups of mice following the PI protocol (Figure 2a). As shown in Figure 2b, liver biopsies of CDAHFD mice exposed to AAA-1s exhibited increased liver macrosteatosis (expressed as % of the total area) when compared to Ctl IgG-recipient mice (93.4% vs. 73.8%, *p* = 0.007). To assess the impact of our PI protocol on chronic low-grade systemic and local inflammation characterizing MASH, Interleukin 6 (IL-6), Interleukin 10 (IL-10), Tumor Necrosis Factor (TNF)-α, and Interleukin 1 (IL-1) β cytokines were measured in plasma, and local inflammation was investigated directly in liver tissue at the transcriptomic level using NanoString^®^ technology.

**Figure 3:**
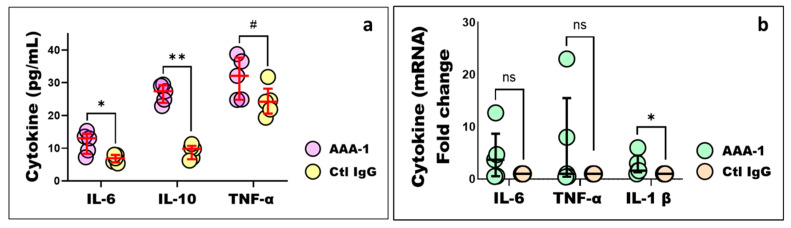
Comparison of inflammatory status between AAA-1-immunized and control IgG-treated mice. (**a**) Effects of AAA-1-immunization on plasma level of cytokines compared to control IgG-treated mice. * *p* = 0.035, ** *p* = 0.007, # *p* = 0.032, M-W test. The median and interquartile range are indicated by red bars. (**b**) Effects of AAA-1 immunization on hepatic production of cytokines mRNAs compared to control IgG-treated mice. The results are expressed as a fold change in AAA-1 values relative to control IgG values. * *p* = 0.04, *p* = 0.68 (*IL-6*), *p* = 0.9 (*TNF-α*), M-W test. Non significant values are denoted as “ns”. Quantification of mRNA transcripts was performed by NanoString technology.

As shown in Figure 3a, higher plasma levels of all these cytokines were observed in AAA-1-immunized mice compared to Ctl IgG-treated mice, except for IL-1β, whose levels were below the detection limit of the assay. These systemic changes were locally accompanied by compatible changes in liver inflammation restricted to *IL-1β* mRNA (Figure 3b). *IL-10* mRNA levels were below the assay detection limit.

Taken together, these data indicate that AAA-1s could directly contribute to the pathogenesis of steatohepatitis by promoting both hepatic steatosis and inflammation.

### 2.2. Elevated Plasma Cytokeratin 18 Levels in AAA-1-Immunized CDAHFD Mice

**Figure 4:**
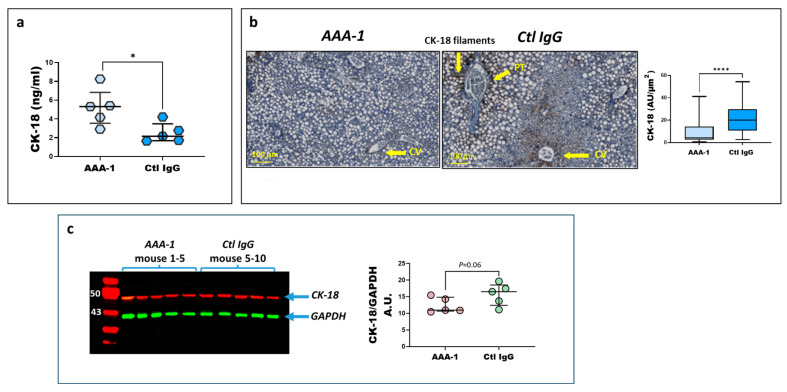
AAA-1s induced a distinct expression of CK-18 in mouse liver tissue compared to the circulating plasma protein levels. (**a**) Mice receiving AAA-1s show higher levels of CK-18 protein compared to those receiving control IgG. * *p* = 0.03, M-W test. (**b**) Representative microphotographs of dissected livers stained for CK-18. Area positive for CK-18 expressed as a percentage and assessed with Qupath software (Belfast, Northern Ireland, version 0.5.0) in ten aleatory selected images. **** *p* < 0.0001, M-W test. Yellow arrows indicate liver structures and CK-18 filaments. (**c**) Western blot analysis of immunized mice liver tissue lysates revealed a trend toward a decrease in CK-18 levels in AAA-1-immunized mice compared to control mice, *p* = 0.06, M-W test. The relative protein expression of CK-18, indicated by the red band, is measured in comparison to the constitutive protein GAPDH, represented by the green band. Abbreviations, PT: portal triad; CV: centrilobular vein; CK-18: Cytokeratin 18.

As a biochemical correlate of cytoskeleton injury underpinning hepatocellular ballooning (the histological hallmark of steatohepatitis), we investigated the effect of PI protocol on CK-18 expression both at the cellular and plasmatic levels. As depicted in Figure 4a, AAA-1-recipient mice exhibited significantly higher plasma levels of CK-18, including intact, apoptosis-, and necrosis-generated fragments, compared to Ctl IgG-treated mice. Conversely, histological analysis of liver sections, shown in Figure 4b, revealed significantly lower levels of intact CK-18 in the liver tissue of AAA-1-immunized mice compared to control mice. Western blot analysis showed a similar and close to significant pattern (Figure 4c). Such a dichotomic pattern was expected due to the inverse relationships reported between systemic and liver CK-18 levels during hepatocyte apoptosis or necrosis.

### 2.3. Impact of AAA-1 Exposure on Liver Fibrosis

**Figure 5:**
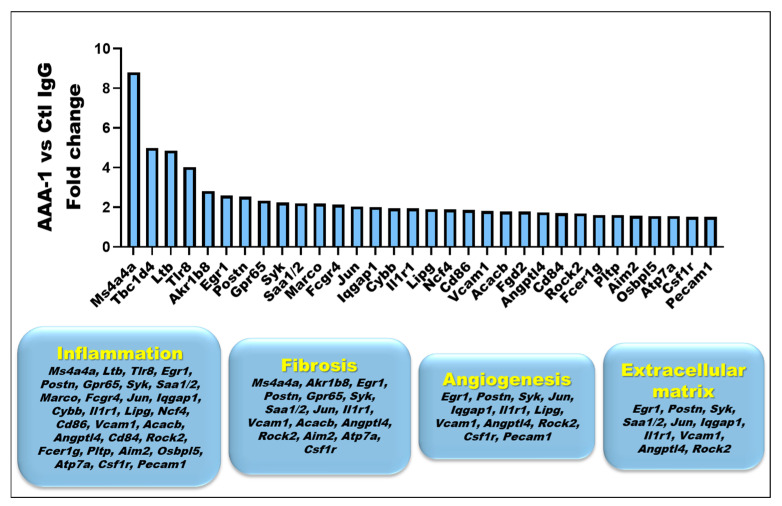
Identification of 32 significantly upregulated genes in AAA-1-immunized mice compared to control IgG-immunized mice through differential gene expression analysis. Total RNA was purified from the cryopreserved liver of CDAHFD mice immunized with AAA-1s or Ctl IgG. Quantification of mRNA transcripts was performed by NanoString nCounter hybridization using 20 ng purified RNA and the pre-designed mouse Fibrosis Profiling Panel (NanoString Technologies, Seattle, WA, USA). A |log fold change| > 1.5 and a *p*-value < 0.05 were defined as differentially expressed mRNAs via R software screening (version 3.56.2, R Foundation for Statistical Computing, Vienna, Austria). The diagram illustrates the gene function even though genes in the inflammation group can indirectly relate to fibrosis, angiogenesis, and extracellular matrix remodeling through their role in immune regulation.

**Figure 6:**
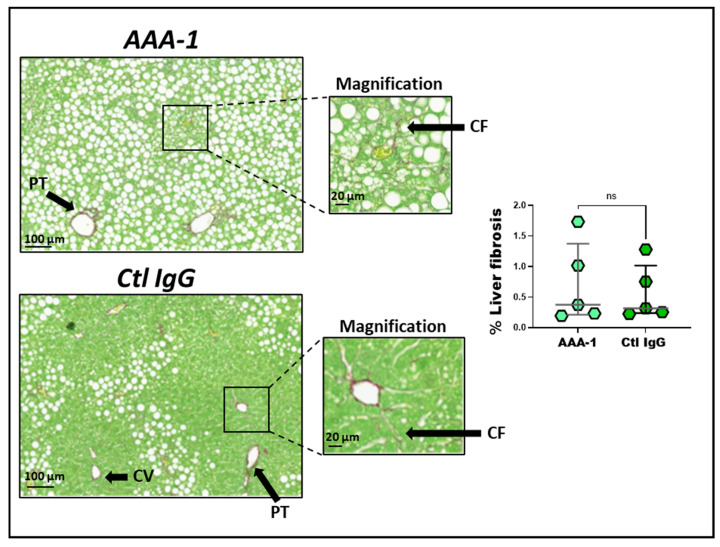
Liver fibrosis levels remain unchanged at the microscopic level. Hepatic fibrosis, marked by excess collagen in the liver, is shown by Sirius red fast green staining where collagen fibers stain bright red while cytoplasmic components stain green. The fibrotic zone is magnified in the right figure. Representative Sirius red fast green staining of liver sections from CDAHFD mice immunized with AAA-1s or control IgG. The results are presented as median and interquartile range. The amount of liver fibrosis was unaffected. *p* = 0.7, M-W test. Non significant values are denoted as “ns”. Arrows indicate liver structures Abbreviations, PT: portal triad; CV: centrilobular vein; CF: collagen fiber.

Due to the impact of our PI protocol on inflammation and CK-18 levels, we intended to capture early signs of fibrosis in the livers of AAA-1-immunized mice through a transcriptomic approach using NanoString^®^ technology allowing the detection of 560 genes involved in inflammation/fibrotic processes together with conventional histology to detect collagen (Sirius red fast green staining). As shown in Figure 5 and Supplemental Table S1, 32 pro-fibrotic transcripts were found to be significantly increased in AAA-1-recipient mice compared to controls, while no differences were observed for classical extracellular matrix-related genes. These upregulated genes are known to span various pathways, including inflammation, angiogenesis, fibrosis, and the non-classical extracellular matrix, as described in the diagram in the caption of Figure 5. No differences were observed for classical extracellular matrix-related genes. However, we could not detect increased collagen at conventional histology using Sirius red fast green staining in mice exposed to AAA-1s, as shown in Figure 6.

## 3. Discussion

The main finding of this work is that AAA-1 PI increases steatohepatitis in CDAHFD mice. Extending previous reports in apoE−/− mice showing that AAA-1s could promote MASLD [27], this study shows that AAA-1s can also contribute to MASH development. Previous works highlighted that AAA-1s could induce MASLD in vitro and in vivo through increased lipogenesis in response to the selective upregulation of sterol regulatory element-binding protein (SREBP)-1 [27]. Additionally, using HepaRG cells, AAA-1s were found to induce a pro-inflammatory response in a Toll-like receptor (TLR)-2-dependent manner [27]. In a similar fashion, mice exposed to AAA-1s had higher plasma cytokine levels compared to the controls (except for IL-1 β levels, which were below the detection range in both mice groups). This observation is in line with the well-documented pro-inflammatory role of AAA-1s retrieved in humans, in vitro, animal studies, and mediated by TLR-2/4 and co-receptor CD14 [26,27,31,32]. The difference observed at the transcription level for the IL-1 β cytokine further confirms the AAA-1 pro-inflammatory effect. Furthermore, as IL-1 β is a major contributor to both chronic liver and atherosclerotic CVD [33,34,35,36], the fact that AAA-1 PI increases *IL-1 β* transcription further supports the hypothesis that these autoantibodies could facilitate MASH development as well.

However, because IL-1 β is a cytokine central to the inflammatory response driving the IL-6 (and TNF-α) pathway [35], knowing why no mRNA expression differences at the local hepatic level could be observed for *IL-6* and *TNF-α*, despite significant differences at the protein plasma levels, warrants further studies. We hypothesize that systemic inflammation driven by immune cells rather than hepatocytes may explain such discrepancy. Factors related to pharmacokinetics due to the intraperitoneal mode of PI used or other ones cannot be currently excluded.

Another compelling evidence for an implication of AAA-1s in steatohepatitis is the impact on circulating and cellular levels of CK-18, characterized by an increase in circulating plasma levels (intact form and fragments) and an intracellular decrease in hepatocytes. By recapitulating the biological signature of hepatocytes ballooning considered as the necessary and sufficient histological hallmark of steatohepatitis [37,38], such observation provides an orthogonal validation of the implication of AAA-1s in MASLD, on top of the effect observed at the cytokine levels.

Moreover, the effects of AAA-1s on plasmatic and hepatic CK-18 levels indicated that these antibodies could also potentially influence liver fibrosis, as elevated CK-18 fragment levels have been strongly associated with the development of liver fibrosis in the context of MASLD and hepatitis C (HCV) [39]. These findings may suggest that CK-18 levels could serve as a reliable marker of liver injury caused by AAA-1s, particularly during the early stages of steatohepatitis when histological changes in the liver might still be minimal. Further investigation is needed to clarify the exact mechanism through which AAA-1s trigger this hepatocellular damage targeting cytoskeletal integrity or activating apoptosis and necrosis through CK-18.

Along this line, our transcriptomic results showed that AAA-1 PI upregulated more than 30 fibrosis-related genes in the liver. The strongest upregulated gene (approximately 8-fold) was membrane-spanning 4-domains, subfamily A, member 4A (*Ms4a4a*). *Ms4A4a* is selectively expressed in immunocompetent cells, such as B cells and macrophages. It is involved in modulating signaling activity associated with various immunoreceptor classes, including pattern recognition receptors (PRRs) and immunoglobulins receptors, and is expressed during macrophage activation [40,41]. Its activation has been ascribed to occur in numerous pathological conditions [41], including systemic sclerosis-associated lung fibrosis in humans [41]. Among the other genes of interest modulated by AAA-1s, Periostin (*POSTN*) was increased by 2.5-fold. This non-structural extracellular matrix protein promotes liver inflammation and fibrosis [42,43] and seems to be a causative agent in multiple fibrotic diseases [44,45]. G-protein-coupled receptor (*GPCR)-65* mRNA was increased by 2.3-fold. This *GPCR* gene plays a crucial role in regulating the progression of liver fibrosis, making it a promising therapeutic target for preventing this condition [46]. Aldo-keto reductase family 1 member B (*AKR1B*)-8 mRNA was increased by 2.8-fold. It is the murine ortholog of *AKR1B10* that is a significantly upregulated gene in the livers of human MASH patients, and its pharmacological inhibitors of *AKR1B8* significantly reduced the pathological features of MASH, such as steatosis, inflammation, and fibrosis in mouse [47]. Serum amyloid A 1/2 (*SAA1/2*) mRNA was increased by 2.2-fold. This acute-phase response protein is known to trigger hepatic steatosis and intrahepatic inflammatory response by forming a SAA1/TLR4/NF-kappaB/SAA1 feedforward regulatory circuit, reported to facilitate MAFLD progression [48]. Vascular cell adhesion molecule-1 (*VCAM-1*) mRNA was also significantly increased, and several works highlight the implication of VCAM-1 in predicting liver fibrosis in MAFLD and progression to MASH [49,50]. However, these changes did not translate into an increase in liver fibrosis upon conventional histology. The reasons for such a discrepancy are elusive but can certainly be explained by the very short PI duration (10 days) and the fact that CDAHFD mice may not be optimal for studying liver fibrosis under a short protocol duration [28,29,30]. AAA-1s may trigger early, pre-fibrotic molecular events in the liver; these molecular changes have not yet been translated into detectable fibrosis, which may require longer exposure or more advanced disease progression to become evident. AAA-1s could prime the liver for fibrosis, potentially offering therapeutic targets for early intervention in liver diseases, like MASLD. Further work is required to clarify this point.

The major limitation of this study lies in the limited number of mice exposed and the short immunization protocol duration prompted by the respect of standard MASH protocols while using CDAHFD mice [30]. We postulate that an extended exposure period could have generated a more pronounced difference in fibrosis markers, but further work is required to confirm or reject this hypothesis. Another limitation of this study is that we did not investigate the molecular mechanisms and pathways underlying the AAA1-induced MASH. However, due to the substantial amount of data pointing to the TLR2-4/CD14 complex mediating all the known deleterious effects of AAA-1s in mice and in vitro [31,32,51,52], we expect similar pathways to be engaged in CDAHFD mice, even if not formally demonstrated. Finally, it is worth mentioning that although H&E staining remains the gold standard for diagnosing hepatic steatosis in clinical practice [53,54,55], we are aware that Oil Red O (ORO) staining for lipids could have been useful in confirming steatosis in mice However, we did not have frozen liver tissue in OCT, which led to a partial loss of tissue architecture and resulted in suboptimal slide preparation and poor ORO staining quality.

In conclusion, our study indicates that AAA-1s can contribute to the pathogenesis of MASH on top of MASLD by promoting systemic and hepatic inflammation. Our transcriptomic observations in hepatic tissue suggest that AAA-1s could directly contribute to liver fibrosis, but they could also indirectly influence the fibrotic process via chronic inflammation, which is a key driver of fibrosis. Further research is needed to elucidate the molecular mechanism underlying the effects of AAA-1s and their possible impact on liver fibrosis. Whether AAA-1s could represent a therapeutic target for managing metabolic liver disease and associated cardiovascular risk remain to be demonstrated.

## 4. Materials and Methods

### 4.1. Animal Experiments

The animal experiment of this study was approved by the Geneva Veterinary Office and the Ethic Commission of Animal Experimentation of the University of Geneva (approval number GE108). Ten-week-old C57/B6J males were purchased from Charles River Laboratories (Les Oncins, France). Ten mice weighing 25–30 g were housed at the animal facility of Geneva University Faculty of Medicine. The mice had unrestricted access to a standard diet and water ad libitum. Following a five-day acclimation period, the mice were randomly divided into two groups and were fed the CDAHFD diet (Research diet#A06071302) for ten days and simultaneously injected with goat polyclonal anti-Apolipoprotein A-1 (Academy Bio-Medical Co., Houston, TX, USA, ref: 11AG2) and respective control IgG (Meridian Life Science, Cincinnati, OH, USA, ref: A66200H), 200 μg per mouse per injection, for ten days, renewing the injection every two days. At the end of ten days, the mice were euthanized, and their livers and plasma were collected for further analysis. Heparinized plasmas were immediately aliquoted and frozen at −80 °C. Livers were immediately embedded in 10% formalin (Sigma-Aldrich, St. Louis, MO, USA) or frozen on dry ice before storage at −80 °C. Fixed tissues were subjected to histological analysis.

### 4.2. Cytokines Assessment

Heparinized plasma was analyzed for IL-6, IL-10, TNF-α, and IL-1 β. Cytokines were measured using the mouse V-Plex Proinflammatory Panel 1 kit from the MesoScale Discovery (MSD) platform (Rockville, MD, USA) on the SQ120 instrument, following the manufacturer’s instructions [56].

### 4.3. Plasma Cytokeratin 18 Assessment

Heparinized plasmas were analyzed for circulating CK-18 concentrations. The M65 ELISA from PEVIVA ELISA kits (TECO medical AG, Sissach, Switzerland) measures soluble CK-18 released from dying cells, including those from necrosis or apoptosis according to their corresponding protocol. Absorbance was measured with the FilterMax F3 Multi-Mode Microplate Reader using SoftMax Pro software (version 7.0.3).

### 4.4. Tissue Staining

Isolated liver tissues embedded in paraffin were cut into 5 μm thick sections and used for different staining. Images were acquired with an automated upright slide scanning microscope in widefield, Zeiss Axio Scan.Z1 (Zeiss, Gottingen, Germany). We used a 20X objective for all images. The images were collected using Axio Scan.Z1 software (version 3.5, Zeiss, Carl Zeiss AG, Oberkochen, Germany). Images after each staining were analyzed using QuPath software (version 0.5.0., Queen’s University Belfast, Northern Ireland) [57].

#### 4.4.1. Steatosis

Steatosis quantification was performed using Hematoxylin–Eosin (H&E) staining, following the standard procedure. Image analysis was performed using QuPath software 0.5.0, and steatosis quantification was based on the regions identified as macrosteatosis. After macrosteatosis regions were identified, the percent area of macrosteatosis in the biopsy image was quantified relative to the total area.

#### 4.4.2. Fibrosis

Fibrosis quantification was achieved using Sirius red fast green (Sigma-Aldrich), where collagen staining appears red on a green background. Image analysis was performed using QuPath software 0.5.0. Fibrosis quantification was realized by selecting ten random fixed areas in each liver image, considering only liver parenchyma to avoid confounding collagen staining of the vessels.

### 4.5. Liver Cytokeratin 18 Assessment

Formalin-fixed paraffin-embedded liver tissue slides were dewaxed and rehydrated using xylene and a series of graded alcohols. After heat-induced antigen retrieval with Tris/EDTA buffer solution (pH 9) (Invitrogen, Waltham, MA, USA), the slides were stained by incubating them with a primary antibody against mouse CK-18 (AbCam, Cambridge, UK; ref. ab181597) diluted 1:800 for 1 h at room temperature. Following three washes with a Reaction Buffer (Ventana Roche, Hoffmann-La Roche, Tucson, AZ, USA), a secondary anti-rabbit antibody conjugated to Horseradish Peroxidase (Dako Agilent EnVision, Santa Clara, CA, USA; ref: K400311-2) was added for 30 min. 3,3′-diaminobenzidine tetrahydrochloride (DAB) substrate was then added for 5 min, and slides were counterstained with H&E. Images were acquired as previously described in the “Tissue Staining” section above, and the area positive for CK-18 staining was assessed with Qupath software 0.5.0 in ten randomly selected sections of each image, considering only the liver parenchyma.

### 4.6. Nanostring Analysis

Total RNA was purified from the liver using TRI Reagent Solution (ThermoFisher Scientific, Waltham, MA, USA). RNA degradation was verified using microcapillary electrophoresis with the Agilent 2100 bio-analyzer in conjunction with the RNA 6000 Nano LabChip kits (Agilent, Santa Clara, CA, USA). The RNA integrity number (RIN) value was approximately equal to 8 for each sample.

Quantification of mRNA transcripts was performed by NanoString nCounter hybridization using 20 ng purified RNA and the pre-designed mouse Fibrosis Profiling Panel (NanoString^®^ Technologies, Seattle, WA, USA) [58,59]. A |log fold change| >1.5 and a *p*-value < 0.05 were defined as differentially expressed mRNAs via R software screening after Benjamini and Hochberg correction (version 3.56.2, Limma package, R Foundation for Statistical Computing, Vienna, Austria)

### 4.7. Western Blot Analysis

The liver lysate was prepared by collecting 100 mg of frozen mouse liver tissue, which was mixed with 500 µL of RIPA buffer (150 mM sodium chloride, 50 mM Tris-HCl, pH 7.4, 1 mM ethylenediaminetetraacetic acid, 1 mM phenylmethylsulfonyl fluoride, 1% Triton X-100, 1% sodium deoxycholate, 0.1% sodium dodecyl sulfate) along with phosphatase and protease inhibitors (Halt Protease & Phosphatase Inhibitor Cocktail, Thermo Fisher Scientific, Waltham, MA, USA). The mixture was then homogenized using a TissueLyser (Qiagen Retsch TissueLyser II, Richmond Scientific, Great Britain) at 25 Hz for 30 s with a 5 mm stainless steel bead (Qiagen, Germantown, MD, USA) added to each tube. Cell debris was removed by centrifugation, and protein concentration was measured using the micro-BCA Protein Assay Kit (Thermo Fisher Scientific, Waltham, MA, USA).

Twenty µg of total protein extract was resolved on a 10% polyacrylamide gel by electrophoresis under reducing conditions, followed by transfer to a polyvinylidene difluoride (PVDF) membrane (Immobilon, Merck-Millipore, Burlington, MA, USA) in the presence of 20% methanol. To reduce nonspecific binding, the membrane was blocked using 5% non-fat milk powder in Tris-buffered saline with Tween 20 (T-TBS) for one hour at room temperature. After blocking, we incubated it with the anti-Cytokeratin 18 antibody (rabbit monoclonal, Abcam, ab181597, Cambridge, MA, USA) diluted 1:2000 in (T-TBS) and 5% of non-fat dry milk, as recommended by the manufacturer, and incubated it overnight at 4 °C. The next day, we washed the membrane six times with T-TBS, added the fluorescent secondary antibody (anti-rabbit, Azure Spectra 700 conjugated, AC2128) (Azure Biosystems, San Diego, CA, USA) diluted 1:10,000, and incubated the membrane for 1 h at room temperature. After this incubation, we washed the membrane six times and incubated it with the anti-GAPDH antibody (goat polyclonal, Abcam, ab157156) and diluted 1:10,000, as suggested by the manufacturer, for 1 h at room temperature. After this incubation, we washed the membrane six times and added the fluorescent anti-goat secondary antibody (Azure Spectra 800 conjugated, AC2157) (Azure Biosystems, San Diego, CA, USA). After a final wash (six times), we performed image acquisition using the Azure 600 Advanced Imaging Systems (Azure biosystems, San Diego, CA, USA). Since the instrument allows capturing images in both green and red channels simultaneously, we obtained an image with the red signal corresponding to CK-18 at the expected size of 47–50 kDa and the green signal corresponding to the loading control GAPDH at 36–40 kDa.

### 4.8. Statistics

All data were statistically analyzed using GraphPad Prism v9.0.2 (GraphPad, San Diego, CA, USA). We use non-parametric tests for all data and the Mann–Whitney test for the difference between two independent groups. A *p*-value of less than 0.05 was considered statistically significant. In the graphs, individual values were plotted with the median and interquartile range. The NanoString results were analyzed using R software (version 3.56.2, R Foundation for Statistical Computing, Vienna, Austria)

The *p*-value was calculated using the unpaired T-test in the limma program package in R language, and the *p*-value was corrected using the Benjamini and Hochberg (BH) method. For each significantly differentially expressed mRNA, a *p*-value < 0.05 and |log fold change| > 0.5 were required.

## Data Availability

The original contributions presented in the study are included in the article/Supplementary Material, further inquiries can be directed to the corresponding author.

## Supplementary Materials

The following supporting information can be downloaded at: https://www.mdpi.com/article/10.3390/ijms252211875/s1. References [60,61,62,63,64,65,66,67,68,69,70,71,72,73,74,75,76,77,78,79,80,81,82,83,84,85,86,87,88,89,90,91,92,93,94,95,96,97] are cited in the supplementary materials.
