# Supplementary material for: Linking Antibodies Against Apolipoprotein A-1 to Metabolic Dysfunction-Associated Steatohepatitis in Mice
Source: Int J Mol Sci. 2024 Nov 5;25(22):11875. doi: 10.3390/ijms252211875 (PMC11594174; doi:10.3390/ijms252211875)
Supplement: Supplementary file 1 [file ijms-25-11875-s001.zip › ijms-3261684-supplementary.pdf]

Supplemental Table S1. Upregulated genes in AAA-1-immunized mice compared to control mice through differential gene expression analysis. A ǀlog fold changeǀ >1.5 and a P-value <0.05 were defined as differentially expressed mRNAs via R software screening. Functional informations are obtained using UniProt databases (https://www.uniprot.org/proteomes/).

Gene	Description	Function	AAA1 vs Ctl IgG Fold change	*P*-value
Ms4a4a	Membrane-spanning 4-domains, subfamily A, member 4A	Receptor. MS4A4A is a tetraspan molecule selectively expressed in macrophages during differentiation and polarization essential for dectin-1-dependent activation of natural killer cell-mediated resistance to metastasis. Its activation is linked to various pathologies, including systemic sclerosis-associated lung fibrosis in humans. [1]	8.80	0.033
Tbc1d4	TBC1 domain family, member 4	May act as a GTPase-activating protein for RAB2A, RAB8A, RAB10 and RAB14. Isoform 2 promotes insulin-induced glucose transporter SLC2A4/GLUT4 translocation at the plasma membrane, thus increasing glucose uptake. [2]	4.99	0.018
Ltb	Lymphotoxin B	Cytokine that binds to LTBR/TNFRSF3. May play a specific role in immune response regulation. [3]	4.85	0.038
Tlr8	Toll-like receptor 8	Endosomal receptor that plays a key role in innate and adaptive immunity. Its activation of downstream transcription factors NF-kappa-B and IRF7 induces pro-inflammatory cytokines and interferons production. [4]	4.02	0.043
Akr1b8	Aldo-keto reductase family 1, member B8	Enzyme with oxidoreductase activity. Role of AKR1B10 and AKR1B8 in the pathogenesis of non-alcoholic steatohepatitis in mouse. It is a significantly upregulated gene in livers of human NASH patients. [5]	2.82	0.011
Egr1	Early growth response 1	Transcriptional regulator of numerous target genes. Regulates the expression of proteins such as IL1B and CXCL2. It has a role in the development of alcohol-induced steatosis. [6]	2.59	0.000
Postn	Periostin, osteoblast specific factor	Plays a role in the formation and remodeling of the extracellular matrix which includes fibronectin. [7] promotes liver inflammation and fibrosis. [8]	2.54	0.001
Gpr65	G-protein coupled receptor 65	Receptor for the glycosphingolipid psychosine and several related glycosphingolipids. Plays a role in immune response. May have a role in activation-induced cell death or differentiation of T-cells. [9] It seems to regulate the progression of liver fibrosis. [10]	2.33	0.010
Syk	Spleen tyrosine kinase	Non-receptor tyrosine kinase which regulates several biological processes including innate and adaptive immunity, cell adhesion, osteoclast maturation, platelet activation and vascular development. [11]	2.24	0.043
Saa1/2	Serum amyloid A1/A2	Major acute-phase response protein. SAA1 has been found to play an important role in lipid metabolism and contributes to bacterial clearance, the regulation of inflammation and tumor pathogenesis. [12] It seems to facilitate NAFLD progression. [13]	2.20	0.020

Marco	Macrophage receptor with collagenous structure	Pattern recognition receptor which is involved in tissue inflammation and immune disorders. [14] MARCO could be a target for the treatment of autoimmune hepatitis. [15]	2.19	0.016
Fcgr4	Fc receptor, IgG, low affinity IV	Binds antigen-IgG complexes during infection, triggering NK cell cytokine production and degranulation to limit viral load. Generates memory-like NK cells that produce high IFNG and eliminate virus-infected cells via ADCC. Regulates NK cell survival and proliferation by preventing progenitor apoptosis. [16]	2.14	0.029
Jun	Jun proto-oncogene	Transcription factor that recognizes and binds to the AP-1. Activation of hepatic JNK, c-Jun, and AP-1 signaling occurred in parallel with the development of steatohepatitis in MCD diet–fed mice. [17]	2.04	0.020
Iqgap1	IQ motif containing GTPase activating protein 1	Acts as a scaffold for organizing a multimolecular complex that links incoming signals to actin cytoskeleton reorganization at the plasma membrane. May also contribute to cell cycle progression after DNA replication arrest. [18] Silencing IQGAP1 alleviates hepatic fibrogenesis via blocking bone marrow mesenchymal stromal cell recruitment to fibrotic liver. [19]	2.01	0.039
Cybb	Cytochrome b-245, beta polypeptide	Critical component of the membrane-bound oxidase of phagocytes that generates superoxide. It participates in the regulation of cellular pH and is blocked by zinc. TAZ/WWTR1-induced Cybb contributes to liver tumor formation in non-alcoholic steatohepatitis. [20]	1.94	0.026
Il1r1	Interleukin 1 receptor, type I	Receptor for IL1A, IL1B and IL1RN. After binding to interleukin-1, it associates with IL1RAP to form a high-affinity receptor complex that activates NF-kappa-B, MAPK, and other pathways. [21] Hepatocyte-specific deletion of IL1-RI reduces liver injury by blocking IL-1-driven autoinflammation, preventing cell death and inflammation in acute liver failure. [22]	1.94	0.008
Lipg	Lipase, endothelial	Endothelial lipase is a member of the triglyceride lipase family and primarily synthesized by vascular endothelial cells. LIPG plays an important role in HDL metabolism and is also involved in cytokine expression and the lipid composition of cells. [23]	1.90	0.027
Ncf4	Neutrophil cytosolic factor 4	NCF4 protein is a subunit of NADPH oxidase, crucial for immune function. NADPH oxidase is active in phagocytes and regulates neutrophils to manage inflammation. Activated hepatic stellate cells have a nonphagocytic NADPH oxidase while phagocytic NADPH oxidase kills bacteria. In chronic liver diseases, both types increase ROS, causing inflammation and fibrogenesis. [24]	1.89	0.032
Cd86	CD86 antigen	It is marker for M1 and M2b macrophages.<br>The origin of hepatic macrophages and the mechanism of macrophage polarization are complex, and their effects on different types of liver diseases and even on different stages of one liver disease are not the same. [25]	1.86	0.034
Vcam1	Vascular cell adhesion molecule 1	Liver sinusoidal endothelial cell-expressed vascular cell adhesion molecule 1 promotes	1.80	0.022

		liver inflammation in nonalcoholic steatohepatitis. During liver injury, liver sinusoidal endothelial cell dysfunction and capillarization promote liver fibrosis. [26]		
Acacb	Acetyl-Coenzyme A carboxylase beta	Enzymes that produce malonyl-CoA for fatty acid synthesis. It is a key enzyme in the *de novo* lipogenesis in the liver. Its inhibition reduces hepatic steatosis in mice by decreasing triglycerides in liver. [27]	1.78	0.007
Fgd2	FYVE, RhoGEF and PH domain containing 2	The protein encoded by this gene belongs to a family of guanine nucleotide exchange factors which control cytoskeleton-dependent membrane rearrangements by activating the cell division cycle 42 (CDC42) protein. [28]	1.78	0.035
Angptl4	Angiopoietin-like 4	ANGPTL4 is a multifaceted secreted protein that is highly expressed in metabolic tissues, most prominently in adipose tissue and liver. It regulates many cellular and physiological functions, mainly via inhibiting lipoprotein lipase activity at the posttranslational level. Importantly, the circulating level of ANGPTL4 is positively correlated with increased risk of cardiovascular disease. [29]	1.74	0.013
Cd84	CD84 antigen	Self-ligand receptor of the signaling lymphocytic activation molecule (SLAM) family. SLAM receptors triggered by homo- or heterotypic cell-cell interactions are modulating the activation and differentiation of a wide variety of immune cells and thus are involved in the regulation and interconnection of both innate and adaptive immune response. [30]	1.70	0.026
Rock2	Rho-associated coiled-coil containing protein kinase 2	ROCK2 regulates key pro-fibrotic pathways involved in both inflammatory reactions and altered extracellular matrix remodelling, [31] implicating this pathway as a potential therapeutic target. [32]	1.69	0.011
Fcer1g	Fc receptor, IgE, high affinity I, gamma polypeptide	Adapter protein containing an immunoreceptor tyrosine-based activation motif (ITAM) that transduces activation signals from various immunoreceptors. As a component of the high-affinity immunoglobulin E (IgE) receptor, mediates allergic inflammatory signaling in mast cells.	1.61	0.040
Pltp	Phospholipid transfer protein	Mediates the transfer of phospholipids and free cholesterol from triglyceride-rich lipoproteins (low density lipoproteins and very low density lipoproteins) into high-density lipoproteins as well as the exchange of phospholipids between triglyceride-rich lipoproteins themselves. [33] It is reported that serum PLTP activity is a risk factor for human cardiovascular disease. [34]	1.61	0.000
Aim2	Absent in melanoma 2	AIM2 is a cytosolic receptor that recognizes double-stranded DNA and triggers the activation of the inflammasome cascade. [35] AIM2 seems to have a role in the pathogenesis of different hepatic diseases, including non-alcoholic fatty liver disease and non-alcoholic steatohepatitis, hepatitis B, liver fibrosis, and hepatocellular carcinoma. [36]	1.57	0.015
Osbpl5	oxysterol binding protein-like 5	OSBP have been implicated in diverse aspects of cellular physiology, including sterol and phospholipid metabolism, vesicle transport, and cell signaling. [37] OSBP regulates hepatic	1.55	0.008

		triglyceride metabolism enhancing hepatic lipogenesis. [38]		
Atp7a	ATPase, Cu++ transporting, alpha polypeptide	Enables P-type divalent copper transporter activity and superoxide dismutase copper chaperone activity. Acts upstream of or within several processes, including animal organ development; cellular biogenic amine metabolic process; and copper ion transport. [39]	1.55	0.004
Csf1r	Colony stimulating factor 1 receptor	CSF-1R also known as macrophage colony-stimulating factor receptor (M-CSF) is an important receptor tyrosine kinase regulating the proliferation, differentiation, and survival of the mononuclear phagocyte lineage cells and macrophages. The excessive activation of CSF-1R may result in inflammation in many organs including liver. [40] M-CSF is potent profibrotic factors in HCV liver fibrosis. [41]	1.52	0.010
Pecam1	Platelet/endothelial cell adhesion molecule 1	PECAM-1 is a protein with adhesive and signaling functions in the immune and the vascular system. Its precise roles in chronic liver inflammation and fibrosis are controversial. [42, 43]	1.51	0.012

References

1. Mattiola, I.; Tomay, F.; De Pizzol, M.; Silva-Gomes, R.; Savino, B.; Gulic, T.; Doni, A.; Lonardi, S.; Astrid Boutet, M.; Nerviani, A.; Carriero, R.; Molgora, M.; Stravalaci, M.; Morone, D.; Shalova, I. N.; Lee, Y.; Biswas, S. K.; Mantovani, G.; Sironi, M.; Pitzalis, C.; Vermi, W.; Bottazzi, B.; Mantovani, A. ;Locati, M. The Macrophage Tetraspan Ms4a4a Enhances Dectin-1-Dependent Nk Cell-Mediated Resistance to Metastasis. *Nat Immunol* **2019**, 20, 1012-1022. 10.1038/s41590-019-0417-y
2. Miinea, C. P.; Sano, H.; Kane, S.; Sano, E.; Fukuda, M.; Peranen, J.; Lane, W. S. ;Lienhard, G. E. As160, the Akt Substrate Regulating Glut4 Translocation, Has a Functional Rab Gtpase-Activating Protein Domain. *Biochem J* **2005**, 391, 87-93. 10.1042/BJ20050887
3. Sudhamsu, J.; Yin, J.; Chiang, E. Y.; Starovasnik, M. A.; Grogan, J. L. ;Hymowitz, S. G. Dimerization of Ltbetar by Ltalpha1beta2 Is Necessary and Sufficient for Signal Transduction. *Proc Natl Acad Sci U S A* **2013**, 110, 19896-19901. 10.1073/pnas.1310838110
4. Qin, J.; Yao, J.; Cui, G.; Xiao, H.; Kim, T. W.; Fraczek, J.; Wightman, P.; Sato, S.; Akira, S.; Puel, A.; Casanova, J. L.; Su, B. ;Li, X. Tlr8-Mediated Nf-Kappab and Jnk Activation Are Tak1-Independent and Mekk3-Dependent. *J Biol Chem* **2006**, 281, 21013-21021. 10.1074/jbc.M512908200
5. Rajak, S.; Gupta, P.; Anjum, B.; Raza, S.; Tewari, A.; Ghosh, S.; Tripathi, M.; Singh, B. K. ;Sinha, R. A. Role of Akr1b10 and Akr1b8 in the Pathogenesis of Non-Alcoholic Steatohepatitis (Nash) in Mouse. *Biochim Biophys Acta Mol Basis Dis* **2022**, 1868, 166319. 10.1016/j.bbadis.2021.166319
6. Thomes, P. G. ;Donohue, T. M. Role of Early Growth Response-1 in the Development of Alcohol-Induced Steatosis. *Curr Mol Pharmacol* **2017**, 10, 179-185. 10.2174/1874467208666150817112529
7. Gillan, L.; Matei, D.; Fishman, D. A.; Gerbin, C. S.; Karlan, B. Y. ;Chang, D. D. Periostin Secreted by Epithelial Ovarian Carcinoma Is a Ligand for Alpha(V)Beta(3) and Alpha(V)Beta(5) Integrins and Promotes Cell Motility. *Cancer Res* **2002**, 62, 5358-5364.
8. Kumar, P.; Smith, T.; Raeman, R.; Chopyk, D. M.; Brink, H.; Liu, Y.; Sulchek, T. ;Anania, F. A. Periostin Promotes Liver Fibrogenesis by Activating Lysyl Oxidase in Hepatic Stellate Cells. *J Biol Chem* **2018**, 293, 12781-12792. 10.1074/jbc.RA117.001601

9. Lassen, K. G.; McKenzie, C. I.; Mari, M.; Murano, T.; Begun, J.; Baxt, L. A.; Goel, G.; Villablanca, E. J.; Kuo, S. Y.; Huang, H.; Macia, L.; Bhan, A. K.; Batten, M.; Daly, M. J.; Reggiori, F.; Mackay, C. R. ;Xavier, R. J. Genetic Coding Variant in Gpr65 Alters Lysosomal Ph and Links Lysosomal Dysfunction with Colitis Risk. *Immunity* **2016**, 44, 1392-1405. 10.1016/j.immuni.2016.05.007
10. Zhang, K.; Zhang, M. X.; Meng, X. X.; Zhu, J.; Wang, J. J.; He, Y. F.; Li, Y. H.; Zhao, S. C.; Shi, Z. M.; Zheng, L. N.; Han, T. ;Hong, W. Targeting Gpr65 Alleviates Hepatic Inflammation and Fibrosis by Suppressing the Jnk and Nf-Kappab Pathways. *Mil Med Res* **2023**, 10, 56. 10.1186/s40779-023-00494-4
11. Wang, L.; Aschenbrenner, D.; Zeng, Z.; Cao, X.; Mayr, D.; Mehta, M.; Capitani, M.; Warner, N.; Pan, J.; Wang, L.; Li, Q.; Zuo, T.; Cohen-Kedar, S.; Lu, J.; Ardy, R. C.; Mulder, D. J.; Dissanayake, D.; Peng, K.; Huang, Z.; Li, X.; Wang, Y.; Wang, X.; Li, S.; Bullers, S.; Gammage, A. N.; Warnatz, K.; Schiefer, A. I.; Krivan, G.; Goda, V.; Kahr, W. H. A.; Lemaire, M.; Genomics England Research, Consortium; Lu, C. Y.; Siddiqui, I.; Surette, M. G.; Kotlarz, D.; Engelhardt, K. R.; Griffin, H. R.; Rottapel, R.; Decaluwe, H.; Laxer, R. M.; Proietti, M.; Hambleton, S.; Elcombe, S.; Guo, C. H.; Grimbacher, B.; Dotan, I.; Ng, S. C.; Freeman, S. A.; Snapper, S. B.; Klein, C.; Boztug, K.; Huang, Y.; Li, D.; Uhlig, H. H. ;Muise, A. M. Gain-of-Function Variants in Syk Cause Immune Dysregulation and Systemic Inflammation in Humans and Mice. *Nat Genet* **2021**, 53, 500-510. 10.1038/s41588-021-00803-4
12. Benditt, E. P.; Hoffman, J. S.; Eriksen, N.; Parmelee, D. C. ;Walsh, K. A. Saa, an Apoprotein of Hdl: Its Structure and Function. *Ann N Y Acad Sci* **1982**, 389, 183-189. 10.1111/j.1749-6632.1982.tb22136.x
13. Jiang, B.; Wang, D.; Hu, Y.; Li, W.; Liu, F.; Zhu, X.; Li, X.; Zhang, H.; Bai, H.; Yang, Q.; Yang, X.; Ben, J. ;Chen, Q. Serum Amyloid A1 Exacerbates Hepatic Steatosis Via Tlr4-Mediated Nf-Kappab Signaling Pathway. *Mol Metab* **2022**, 59, 101462. 10.1016/j.molmet.2022.101462
14. Elomaa, O.; Sankala, M.; Pikkarainen, T.; Bergmann, U.; Tuuttila, A.; Raatikainen-Ahokas, A.; Sariola, H. ;Tryggvason, K. Structure of the Human Macrophage Marco Receptor and Characterization of Its Bacteria-Binding Region. *J Biol Chem* **1998**, 273, 4530-4538. 10.1074/jbc.273.8.4530
15. Cai, T.; Xu, L.; Xia, D.; Zhu, L.; Lin, Y.; Yu, S.; Zhu, K.; Wang, X.; Pan, C.; Chen, Y. ;Chen, D. Polyguanine Alleviated Autoimmune Hepatitis through Regulation of Macrophage Receptor with Collagenous Structure and Tlr4-Trif-Nf-Kappab Signalling. *J Cell Mol Med* **2022**, 26, 5690-5701. 10.1111/jcmm.17599
16. DiLillo, D. J.; Tan, G. S.; Palese, P. ;Ravetch, J. V. Broadly Neutralizing Hemagglutinin Stalk-Specific Antibodies Require Fcgammar Interactions for Protection against Influenza Virus in Vivo. *Nat Med* **2014**, 20, 143-151. 10.1038/nm.3443
17. Schattenberg, J. M.; Singh, R.; Wang, Y.; Lefkowitch, J. H.; Rigoli, R. M.; Scherer, P. E. ;Czaja, M. J. Jnk1 but Not Jnk2 Promotes the Development of Steatohepatitis in Mice. *Hepatology* **2006**, 43, 163-172. 10.1002/hep.20999
18. Johnson, M.; Sharma, M.; Brocardo, M. G. ;Henderson, B. R. Iqgap1 Translocates to the Nucleus in Early S-Phase and Contributes to Cell Cycle Progression after DNA Replication Arrest. *Int J Biochem Cell Biol* **2011**, 43, 65-73. 10.1016/j.biocel.2010.09.014
19. Ma, Y.; Chang, N.; Liu, Y.; Liu, F.; Dong, C.; Hou, L.; Qi, C.; Yang, L. ;Li, L. Silencing Iqgap1 Alleviates Hepatic Fibrogenesis Via Blocking Bone Marrow Mesenchymal Stromal Cell Recruitment to Fibrotic Liver. *Mol Ther Nucleic Acids* **2022**, 27, 471-483. 10.1016/j.omtn.2021.12.020
20. Wang, X.; Zeldin, S.; Shi, H.; Zhu, C.; Saito, Y.; Corey, K. E.; Osganian, S. A.; Remotti, H. E.; Verna, E. C.; Pajvani, U. B.; Schwabe, R. F. ;Tabas, I. Taz-Induced Cybb Contributes to Liver Tumor Formation in Non-Alcoholic Steatohepatitis. *J Hepatol* **2022**, 76, 910-920. 10.1016/j.jhep.2021.11.031
21. Wang, Y.; Wang, J.; Zheng, W.; Zhang, J.; Wang, J.; Jin, T.; Tao, P.; Wang, Y.; Liu, C.; Huang, J.; Lee, P. Y.; Yu, X. ;Zhou, Q. Identification of an Il-1 Receptor Mutation Driving

Autoinflammation Directs Il-1-Targeted Drug Design. *Immunity* **2023**, 56, 1485-1501 e1487. 10.1016/j.immuni.2023.05.014

22. Wang, C.; Deng, L.; Hong, M.; Akkaraju, G. R.; Inoue, J. ;Chen, Z. J. Tak1 Is a Ubiquitin-Dependent Kinase of Mkk and Ikk. *Nature* **2001**, 412, 346-351. 10.1038/35085597
23. Yu, J. E.; Han, S. Y.; Wolfson, B. ;Zhou, Q. The Role of Endothelial Lipase in Lipid Metabolism, Inflammation, and Cancer. *Histol Histopathol* **2018**, 33, 1-10. 10.14670/HH-11-905
24. De Minicis, S.; Bataller, R. ;Brenner, D. A. Nadph Oxidase in the Liver: Defensive, Offensive, or Fibrogenic? *Gastroenterology* **2006**, 131, 272-275. 10.1053/j.gastro.2006.05.048
25. Wang, C.; Ma, C.; Gong, L.; Guo, Y.; Fu, K.; Zhang, Y.; Zhou, H. ;Li, Y. Macrophage Polarization and Its Role in Liver Disease. *Front Immunol* **2021**, 12, 803037. 10.3389/fimmu.2021.803037
26. Hintermann, E. ;Christen, U. The Many Roles of Cell Adhesion Molecules in Hepatic Fibrosis. *Cells* **2019**, 8, 10.3390/cells8121503
27. Kim, C. W.; Addy, C.; Kusunoki, J.; Anderson, N. N.; Deja, S.; Fu, X.; Burgess, S. C.; Li, C.; Ruddy, M.; Chakravarthy, M.; Previs, S.; Milstein, S.; Fitzgerald, K.; Kelley, D. E. ;Horton, J. D. Acetyl Coa Carboxylase Inhibition Reduces Hepatic Steatosis but Elevates Plasma Triglycerides in Mice and Humans: A Bedside to Bench Investigation. *Cell Metab* **2017**, 26, 394-406 e396. 10.1016/j.cmet.2017.07.009
28. Huber, C.; Martensson, A.; Bokoch, G. M.; Nemazee, D. ;Gavin, A. L. Fgd2, a Cdc42-Specific Exchange Factor Expressed by Antigen-Presenting Cells, Localizes to Early Endosomes and Active Membrane Ruffles. *J Biol Chem* **2008**, 283, 34002-34012. 10.1074/jbc.M803957200
29. Aryal, B.; Price, N. L.; Suarez, Y. ;Fernandez-Hernando, C. Angptl4 in Metabolic and Cardiovascular Disease. *Trends Mol Med* **2019**, 25, 723-734. 10.1016/j.molmed.2019.05.010
30. Martin, M.; Romero, X.; de la Fuente, M. A.; Tovar, V.; Zapater, N.; Esplugues, E.; Pizcueta, P.; Bosch, J. ;Engel, P. Cd84 Functions as a Homophilic Adhesion Molecule and Enhances Ifn-Gamma Secretion: Adhesion Is Mediated by Ig-Like Domain 1. *J Immunol* **2001**, 167, 3668-3676. 10.4049/jimmunol.167.7.3668
31. Hoon, J. L.; Tan, M. H. ;Koh, C. G. The Regulation of Cellular Responses to Mechanical Cues by Rho Gtpases. *Cells* **2016**, 5, 10.3390/cells5020017
32. Nalkurthi, C.; Schroder, W. A.; Melino, M.; Irvine, K. M.; Nyuydzefe, M.; Chen, W.; Liu, J.; Teng, M. W. L.; Hill, G. R.; Bertolino, P.; Blazar, B. R.; Miller, G. C.; Clouston, A. D.; Zanin-Zhorov, A. ;MacDonald, K. P. A. Rock2 Inhibition Attenuates Profibrogenic Immune Cell Function to Reverse Thioacetamide-Induced Liver Fibrosis. *JHEP Rep* **2022**, 4, 100386. 10.1016/j.jhepr.2021.100386
33. Oka, T.; Kujiraoka, T.; Ito, M.; Egashira, T.; Takahashi, S.; Nanjee, M. N.; Miller, N. E.; Metso, J.; Olkkonen, V. M.; Ehnholm, C.; Jauhiainen, M. ;Hattori, H. Distribution of Phospholipid Transfer Protein in Human Plasma: Presence of Two Forms of Phospholipid Transfer Protein, One Catalytically Active and the Other Inactive. *J Lipid Res* **2000**, 41, 1651-1657.
34. Schlitt, A.; Bickel, C.; Thumma, P.; Blankenberg, S.; Rupprecht, H. J.; Meyer, J. ;Jiang, X. C. High Plasma Phospholipid Transfer Protein Levels as a Risk Factor for Coronary Artery Disease. *Arterioscler Thromb Vasc Biol* **2003**, 23, 1857-1862. 10.1161/01.ATV.0000094433.98445.7F
35. Hornung, V.; Ablasser, A.; Charrel-Dennis, M.; Bauernfeind, F.; Horvath, G.; Caffrey, D. R.; Latz, E. ;Fitzgerald, K. A. Aim2 Recognizes Cytosolic Dsdna and Forms a Caspase-1-Activating Inflammasome with Asc. *Nature* **2009**, 458, 514-518. 10.1038/nature07725
36. Lozano-Ruiz, B. ;Gonzalez-Navajas, J. M. The Emerging Relevance of Aim2 in Liver Disease. *Int J Mol Sci* **2020**, 21, 10.3390/ijms21186535
37. Perry, R. J. ;Ridgway, N. D. Oxysterol-Binding Protein and Vesicle-Associated Membrane Protein-Associated Protein Are Required for Sterol-Dependent Activation of the Ceramide Transport Protein. *Mol Biol Cell* **2006**, 17, 2604-2616. 10.1091/mbc.e06-01-0060
38. Yan, D.; Lehto, M.; Rasilainen, L.; Metso, J.; Ehnholm, C.; Yla-Herttuala, S.; Jauhiainen, M. ;Olkkonen, V. M. Oxysterol Binding Protein Induces Upregulation of Srebp-1c and Enhances

Hepatic Lipogenesis. *Arterioscler Thromb Vasc Biol* **2007**, 27, 1108-1114. 10.1161/ATVBAHA.106.138545

39. Vulpe, C.; Levinson, B.; Whitney, S.; Packman, S. ;Gitschier, J. Isolation of a Candidate Gene for Menkes Disease and Evidence That It Encodes a Copper-Transporting Atpase. *Nat Genet* **1993**, 3, 7-13. 10.1038/ng0193-7
40. Xiang, C.; Li, H. ;Tang, W. Targeting Csf-1r Represents an Effective Strategy in Modulating Inflammatory Diseases. *Pharmacol Res* **2023**, 187, 106566. 10.1016/j.phrs.2022.106566
41. Preisser, L.; Miot, C.; Le Guillou-Guillemette, H.; Beaumont, E.; Foucher, E. D.; Garo, E.; Blanchard, S.; Fremaux, I.; Croue, A.; Fouchard, I.; Lunel-Fabiani, F.; Boursier, J.; Roingeard, P.; Cales, P.; Delneste, Y. ;Jeannin, P. Il-34 and Macrophage Colony-Stimulating Factor Are Overexpressed in Hepatitis C Virus Fibrosis and Induce Profibrotic Macrophages That Promote Collagen Synthesis by Hepatic Stellate Cells. *Hepatology* **2014**, 60, 1879-1890. 10.1002/hep.27328
42. Couvelard, A.; Scoazec, J. Y. ;Feldmann, G. Expression of Cell-Cell and Cell-Matrix Adhesion Proteins by Sinusoidal Endothelial Cells in the Normal and Cirrhotic Human Liver. *Am J Pathol* **1993**, 143, 738-752.
43. Goel, R.; Boylan, B.; Gruman, L.; Newman, P. J.; North, P. E. ;Newman, D. K. The Proinflammatory Phenotype of Pecam-1-Deficient Mice Results in Atherogenic Diet-Induced Steatohepatitis. *Am J Physiol Gastrointest Liver Physiol* **2007**, 293, G1205-1214. 10.1152/ajpgi.00157.2007
